# The effect of deadlines on cancer screening completion: a randomized controlled trial

**DOI:** 10.1038/s41598-021-93334-1

**Published:** 2021-07-06

**Authors:** Alicea Lieberman, Ayelet Gneezy, Emily Berry, Stacie Miller, Mark Koch, Keith E. Argenbright, Samir Gupta

**Affiliations:** 1 Anderson School of Management, University of California, Los Angeles, Los Angeles, CA USA; 2 Rady School of Management, University of California, San Diego, La Jolla, CA USA; 3 University of Texas Southwestern Medical Center, Moncrief Cancer Institute, Fort Worth, TX USA; 4 Department of Family Medicine, John Peter Smith Health Network, Fort Worth, TX USA; 5 University of Texas Southwestern Medical Center Harold C. Simmons Cancer Center, Dallas, TX USA; 6 Department of Population and Data Sciences, University of Texas Southwestern Medical Center, Dallas, TX USA; 7 San Diego Veterans Affairs Healthcare System, San Diego, CA USA; 8 Department of Internal Medicine, Division of Gastroenterology, and the Moores Cancer Center, University of California San Diego, San Diego, CA USA

**Keywords:** Cancer screening, Population screening, Human behaviour

## Abstract

Cancer is the second leading cause of death in the United States. Although screening facilitates prevention and early detection and is one of the most effective approaches to reducing cancer mortality, participation is low—particularly among underserved populations. In a large, preregistered field experiment (*n* = 7711), we tested whether deadlines—both with and without monetary incentives tied to them—increase colorectal cancer (CRC) screening. We found that all screening invitations with an imposed deadline increased completion, ranging from 2.5% to 7.3% relative to control (*p*s < .004). Most importantly, individuals who received a *short* deadline with no incentive were as likely to complete screening (9.7%) as those whose invitation included a deadline coupled with either a small (9.1%) or large declining financial incentive (12.0%; *p*s = .57 and .04, respectively). These results suggest that merely imposing deadlines—especially short ones—can significantly increase CRC screening completion, and may also have implications for other forms of cancer screening.

## Introduction

Over 38% of people in the U.S. are expected to be diagnosed with cancer in their lifetime^1^. Screening facilitates early detection and prevention, making it one of the most effective tools for reducing cancer mortality. However, participation in cancer screening is suboptimal^2^. Building on research in the social sciences, we designed a large field experiment testing interventions aimed to increase cancer screening, focusing on colorectal cancer (CRC)—the second leading cause of cancer death in the U.S.^3,4^.

Following the U.S. Preventive Services Task Force recommendation that adults aged 50–75 participate in CRC screening^5^, researchers and practitioners have expended extensive efforts to promote screening completion, including the development of more accessible forms of screening such as fecal immunochemical tests (FITs). FIT is a non-invasive self-collected stool test that individuals can complete in the privacy of their own home. Yet, despite widespread availability of multiple CRC screening options (e.g., colonoscopy, FIT), participation remains suboptimal^6–9^. In 2018, only 67% of age-eligible adults were up-to-date with colorectal cancer screening; and, proportion up-to-date was even lower for multiple racial ethnic groups, such as Hispanics (59%), American Indian/Alaska Natives (56%), and Asians (58%; 10).

Qualitative investigations on the barriers to FIT completion have shown that even though FITs offer a relatively accessible form of screening (compared to more invasive tests), they are still perceived as aversive^11–13^. Patients’ top-cited reasons for non-completion include negative emotions associated with the task (e.g., discomfort, disgust, fear) and procrastination tendencies^11–13^. These insights are consistent with research findings in a variety of disciplines showing that when faced with aversive tasks, people often procrastinate^14–18^.

Procrastination—"the voluntary delay of an intention despite the expectation of being worse off for the delay”^19^—has been both directly and indirectly linked to poor health outcomes^16,17,20^. While researchers have identified a variety of factors that promote procrastination, a common theme is the perceived disconnect between one’s present and future selves, with pronounced focus on the former^16,18,21,22^. This disconnect often leads people to prefer options that satisfy their present (vs. future) self, a preference that is further accentuated with tasks involving immediate costs and/or delayed rewards^15,19,23^. Being a strategy that aims to detect, as well as prevent cancer, most patients are asymptomatic at the time of CRC screening. As a result, the benefits of completing screening (i.e., early detection) are temporally remote, whereas the unpleasantness associated with it is quite salient. Further, individuals often discount future efforts of a task more heavily than the benefits associated with it, consequently increasing the attractiveness of delaying it to a later time^24–26^.

Although there exist many reasons why an individual might fail to complete FIT—even if willing and motivated (e.g., access to care, costs, comorbidities, preference for a different test;^11–13,27^)—evidence from research on the drivers of procrastination^16–19,21,22,25,26,28^ and on reported barriers to FIT completion^11–13^, highlights procrastination as a potentially important barrier. Moreover, because procrastination often involves repeated postponement of a behavior^21^, it may result in failure to complete FIT altogether.

Mounting evidence demonstrates that procrastination can be attenuated by the imposition of deadlines (i.e., constraining the allowable time window for task completion; 18, 29, 30), arguably because motivation increases at a hyperbolic rate as the deadline approaches^31,32^. Deadlines have been shown to effectively curb procrastination across a range of behaviors, including making changes to retirement savings plans^14^, signing up for health plans^18^, redeeming gift cards and rebates^26^, making product returns^33^, and visiting museums^18^. Further, past research shows that shorter deadlines are even more effective than longer ones^18,33^. Thus, in addition to testing whether offering FIT invitations with deadlines impacted completion relative to an invitation with no deadline, we also tested whether shorter deadlines would be more effective than longer ones.

Finally, we tested whether coupling deadlines with a financial incentive would further enhance their effectiveness. Financial incentives are commonly used in efforts to motivate behavior^34^. The popularity of financial incentives among researchers and practitioners has spiked in recent years, leading to their integration in programs aimed to promote a variety of health behaviors, including exercise, smoking cessation, healthy eating, and cancer screening^35–38^. Attempts to increase colorectal cancer screening using financial incentives offer mixed results, with some studies suggesting they are effective^39,40^, while others find they are not^8,41^. For instance, a recent study found that offering a modest incentive ($5 or $10) did not influence behavior relative to non-incentivized invitations^8,41^. Similarly, another study found that neither fixed incentives ($5, $10, or $20) nor an entry into a lottery for $500 improved participation over usual care, whereas a 1 in 10 chance of receiving $50 did^42^. The fact that some incentives successfully promote CRC screening whereas others do not may be explained by research showing that subtle changes to the features of an incentive—e.g., when it is offered, how it is framed—can influence its effectiveness^34^.

Here, we test the impact of declining financial incentives on FIT completion relative to non-financially incentivized deadlines and to standard outreach (i.e., control). Unlike fixed incentives—offering a fixed reward for task completion—declining incentives offer monetary rewards that decrease over time, such that sooner completion is linked with a higher reward. Declining incentives leverage past research, in several disciplines, showing that individuals are loss averse^43,44^. In the context of our investigation, loss aversion suggests that when presented with a reward schedule that decreases over time, patients should want to avoid “losing” the larger, sooner reward, motivating them to complete the test before the first deadline expires^44^. This proposition is also supported by recent findings suggesting that by tapping into individuals’ desire to avoid experiencing regret, declining incentives have the potential to encourage targeted behaviors^45^. Lastly, to examine how declining incentives influence behavior, we tested two levels of declining incentives, allowing us to assess whether the reward size matters under this incentive structure.

## The present research

**Figure 1:**
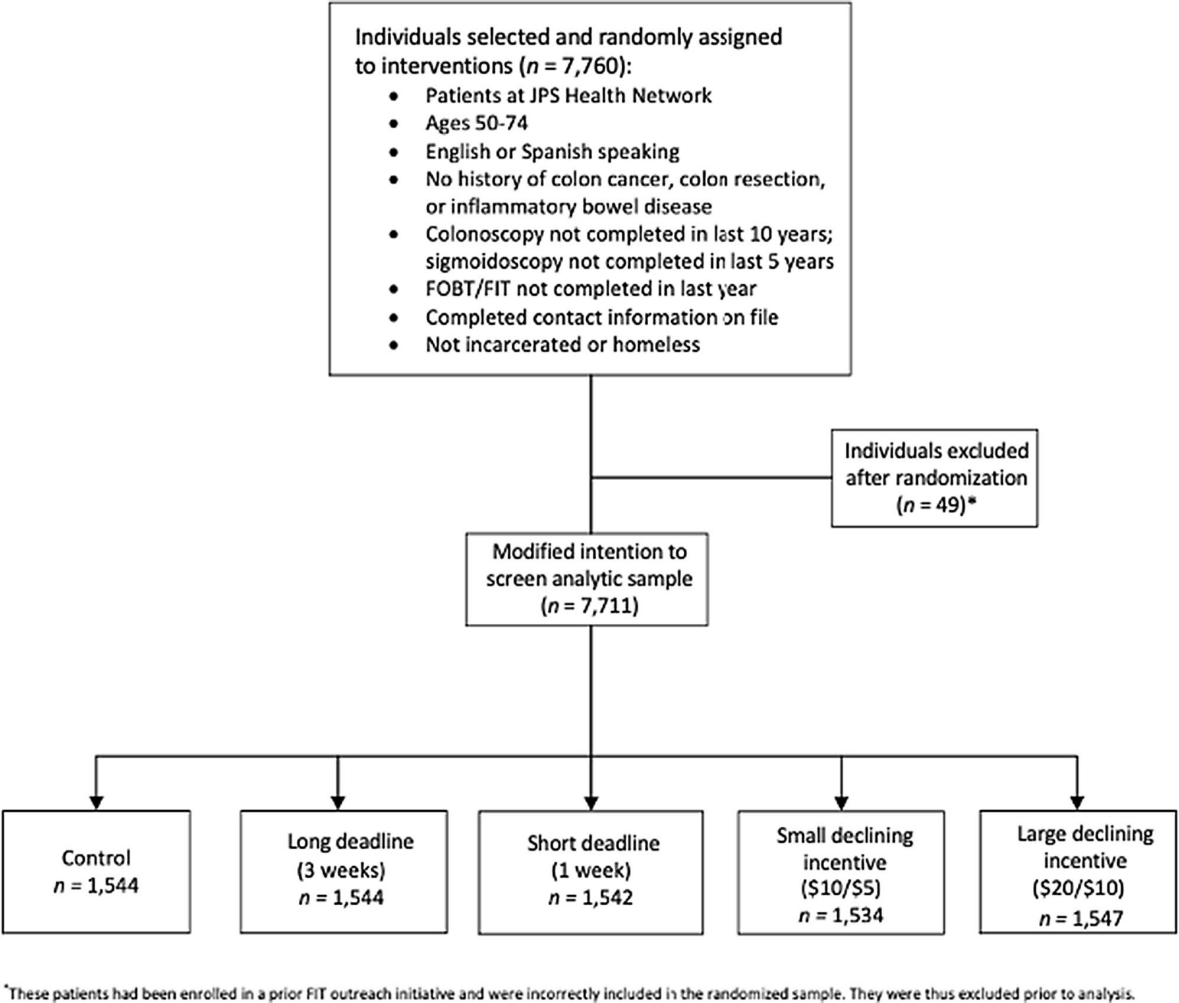
Study flow.

We designed our field experiment to test the effectiveness of short and long deadlines on FIT completion, as well as the effects of declining financial incentives. Prior to data collection, we preregistered our field experiment on ClinicalTrials.gov (ClinicalTrials.gov Identifier: NCT03181334; Date of first registration: 08/06/2017). We invited individuals to complete a mailed FIT screening either with a standard invitation or one of four treatment invitations. Specifically, 7711 individuals from a large safety-net health network were randomly assigned to receive one of the following five FIT invitations: (1) control (standard outreach; *n* = 1544); (2) long deadline (3 weeks; *n* = 1544); (3) short deadline (1 week; *n* = 1542); (4) small declining incentive ($10 for completion within 1 week or $5 for completion within 3 weeks; *n* = 1534); and (5) large declining incentive ($20 for completion within 1 week or $10 for completion within 3 weeks; *n* = 1547) (Fig. 1). Sample size was based on our preregistered power analysis, in which we estimated needing 1026 participants per condition to achieve 80% power to detect at least an 8% difference between groups when using a chi-square test with a two-sided Bonferroni corrected significance level of 0.005 (= 0.05/10). To remain consistent with our preregistration, we report significance as the Bonferroni corrected significance level of *p* ≤ 0.005.

Our preregistered primary dependent variable is the proportion of participants who completed the FIT within 3 weeks of mailing. We also report secondary analyses examining completion likelihood within 1 week of invitation, average time to completion, and completion likelihood within 1 year. The design of our study allowed us to test (1) whether imposing any deadline increased completion relative to a standard invitation, (2) whether a shorter deadline was more effective than a longer deadline, (3) whether deadlines coupled with declining incentives increased completion above and beyond deadlines with no financial incentives tied to them, and (4) whether the magnitude of the declining incentive mattered. We predicted that offering a FIT with any deadline would increase completion relative to a FIT invitation without a deadline, and that the shorter deadline would be more effective than the longer deadline. We further predicted that coupling the deadlines with declining financial incentives would motivate completion above and beyond a non-financially incentivized deadline. Finally, we posited that the incentivized-deadline conditions would not differ from one another, because behavior would be driven primarily by patients’ motivation to avoid losing the larger incentive associated with early completion^44^ rather than the face value of the incentive.

## Results

### Demographics

Participants had a mean (sd) age of 58 years (5.1), 62% were female, 39.9% were white or Caucasian, 27.7% were black or African American, 30.3% were Hispanic or Latino, and 18.4% reported Spanish as their primary language (Table 1). Participant characteristics were not significantly different across conditions.

**Table 1:** Participant characteristics at baseline.

	Control	Long deadline (3 weeks)	Short deadline (1 week)	Small declining ($10/$5)	Large declining ($20/$10)
	*n* = 1544	*n* = 1544	*n* = 1542	*n* = 1534	*n* = 1547
**Characteristic (%)**					
Female sex, no. (%)	945 (61.2)	965 (62.5)	955 (61.9)	963 (62.8)	953 (61.6)
**Race, n (%)**^**1**^					
White	621 (40.3)	606 (39.3)	634 (41.1)	606 (39.6)	605 (39.1)
Black	417 (27.1)	430 (27.9)	411 (26.7)	430 (28.1)	444 (28.7)
Unknown	503 (32.6)	507 (32.9)	496 (32.2)	496 (32.4)	498 (32.2)
**Ethnicity, n (%)**					
Hispanic or Latino	463 (30.0)	483 (31.3)	479 (31.1)	476 (31.1)	433 (28.0)
Not Hispanic or Latino	1073 (69.7)	1050 (68.1)	1051 (68.2)	1048 (68.4)	1101 (71.2)
Unknown	4 (2.6)	9 (5.8)	10 (6.5)	8 (5.2)	13 (8.4)
**Language, n (%)**					
English	1269 (82.2)	1241 (80.4)	1261 (81.8)	1248 (81.4)	1277 (82.5)
Spanish	275 (17.8)	303 (19.6)	281 (18.2)	286 (18.6)	270 (17.5)
Age (years), mean (sd)	58.1 (5.1)	57.8 (5.0)	57.9 (5.1)	58.0 (5.1)	58.0 (5.1)

^1^Seven participants had no entry for the race variable, and 10 participants either had no entry or refused to provide ethnicity information.

^2^The declining-incentive conditions included incentivized deadlines: small declining incentive ($10 for completion within 1 week or $5 for completion within 3 weeks) and large declining incentive ($20 for completion within 1 week or $10 for completion within 3 weeks).

### FIT completion within 3 weeks

Table 2 presents the results of our primary dependent variable: the proportion of individuals who completed the FIT within 3 weeks of invitation. FIT completion was significantly different across conditions ($\chi ^{2}$(4, *n* = 7711) = 59.70, *p* < 0.001, ϕ = 0.09). Completion rates were significantly higher in all four treatment conditions (9.7% short deadline, 7.2% long deadline, 9.1% small declining, and 12.0% large declining) relative to control (4.7%, *ps* < 0.004).

**Table 2:** FIT completion within 3 weeks.

	Control	Long deadline (3 weeks)	Short deadline (1 week)	Small declining ($10/$5)	Large declining ($20/$10)
	*n* = 1544	*n* = 1544	*n* = 1542	*n* = 1534	*n* = 1547
FIT completion (%) [95% CI]	4.7 [3.7–5.8]	7.2 [5.9–8.5]	9.7 [8.2–11.2]	9.1 [7.7–10.6]	12.0 [10.4–13.6]
Difference versus control (%)	–	2.5	5.0	4.4	7.3
*P* value		0.004	< 0.001	< 0.001	< 0.001
Difference versus long deadline (%)	–	–	2.5	1.9	4.8
*P* value			0.01	0.05	< 0.001
Difference versus short deadline (%)	–	–	–	− 0.06	2.3
*P* value	–	–	–	0.57	0.04
Difference versus small declining (%)	–	–	–	–	2.9
*P* value					0.009

^1^The declining-incentive conditions included incentivized deadlines: small declining incentive ($10 for completion within 1 week or $5 for completion within 3 weeks) and large declining incentive ($20 for completion within 1 week or $10 for completion within 3 weeks).

Further analyses revealed completion was marginally higher in response to the short deadline than in response to the long deadline ($\chi ^{2}$ (1, *n* = 3086) = 6.42, *p* = 0.011, ϕ = 0.05). Most interestingly, FIT completion in the short-deadline condition was not statistically different than completion rates in the small-declining ($\chi ^{2}$ (1, *n* = 3076) = 0.33, *p* = 0.568, ϕ = 0.01) or large-declining conditions ($\chi ^{2}$ (1, *n* = 3089) = 4.20, *p* = 0.04, ϕ = 0.04), suggesting a short deadline was as effective at motivating FIT completion as a deadline that included a financial incentive.

Finally, completion was greater in the large-declining condition than in the long-deadline condition ($\chi ^{2}$ (1, *n* = 3091) = 20.79, *p* < 0.001, ϕ = 0.08), and the small-declining condition ($\chi ^{2}$ (1, *n* = 3081) = 6.83 *p* = 0.009, ϕ = 0.05).

### FIT completion within 1 week

Secondary analyses revealed that of participants completing the FIT within 3 weeks, the majority (72.4%) did so within the first week. Completion within the first week differed across conditions ($\chi ^{2}$ (4, *n* = 660) = 9.63, *p* = 0.047, ϕ = 0.12; see Table 3). Relative to control (64.4%), participants in the short-deadline condition were more likely to complete the FIT within the first week (79.3%; $\chi ^{2}$ (1, *n* = 223) = 5.77, *p* = 0.016, ϕ = 0.16). We found no difference in first-week-completion likelihood among individuals in the long-deadline (64.9%), small-declining (72.1%), or large-declining (74.7%) conditions relative to control (*ps* = 0.947, 0.243, and 0.096, respectively). Participants in the short-deadline condition were also more likely to return the kit within the first week than participants in the long-deadline condition ($\chi ^{2}$ (1, *n* = 261) = 6.80, *p* = 0.009, ϕ = 0.16). No other comparisons were significant (*ps* > 0.07).

**Table 3:** FIT completion within 1 week, conditional on completion within 3 weeks.

	Control	Long deadline (3 weeks)	Short deadline (1 week)	Small declining ($10/$5)	Large declining ($20/$10)
Number of participants who completed FIT within three weeks	*n* = 73	*n* = 111	*n* = 150	*n* = 140	*n* = 186
FIT completion [95% CI]	64.4 [53.1,75.6]	64.9 [55.8,73.9]	79.3 [72.8, 85.9]	72.1 [64.6, 79.7]	74.7 [68.4, 81.0]
Difference versus control	–	0.5	14.9	7.8	10.3
*P* value		0.947	0.016	0.243	0.096
Difference versus long deadline	–	–	14.5	7.3	9.9
*P* value			0.009	0.216	0.070
Difference versus short deadline	–	–	–	− 7.2	− 4.6
*P* value	–	–	–	0.153	0.321
Difference versus small declining	–	–	–	–	2.6
*P* value					0.60

^1^The declining incentive conditions included incentivized deadlines: small declining incentive ($10 for completion within 1 week or $5 for completion within 3 weeks) and large declining incentive ($20 for completion within 1 week or $10 for completion within 3 weeks).

### Average time to completion

Among participants who completed the FIT within the prespecified 3-week period, the average number of days between invitation and completion was 8.37. Using a Kruskal–Wallis H test, we found completion times differed significantly across conditions ($\chi ^{2}$ (4, *n* = 660) = 12.01, *p* = 0.017, ε^2^ = 0.02). A post-hoc pairwise comparison using an adjusted Wilcoxon rank sum test indicated participants in the large-declining condition (7.66 days) completed the FIT, on average, sooner than participants in the control (9.81 days; *p* = 0.052) and in the long-deadline (10 days; *p* = 0.052) conditions. No other comparisons were statistically significant (*p*s ≥ 0.069).

### FIT completion within 1 year of invitation

Secondary analyses comparing FIT completion within 1 year of invitation revealed a similar pattern. Consistent with the primary analysis, completion was significantly different across conditions ($\chi ^{2}$(4, *n* = 7711) = 41.20, *p* < 0.001, ϕ = 0.07). More specifically, relative to control (9.1%), completion was significantly higher in the short-deadline (14.1%), small-declining (13.9%), and large-declining (16.2%) conditions (*ps* < 0.001); however, the difference between control and the long-deadline condition did not reach significance (11.5%; $\chi ^{2}$ (1, *n* = 3088) = 5.06, *p* = 0.024, ϕ = 0.04).

Difference in completion within 1 year between participants in the short-deadline and long-deadline condition did not reach statistical significance ($\chi ^{2}$ (1, *n* = 3086) = 4.69, *p* = 0.030, ϕ = 0.04). Relative to the short-deadline condition, completion rates were not statistically different in the small-declining (*p* = 0.840) or large-declining conditions (*p* = 0.106). Participants in the large-declining condition were, however, more likely to complete the FIT than participants in the long-deadline condition ($\chi ^{2}$ (1, *n* = 3091) = 14.26, *p* < 0.001, ϕ = 0.07). Finally, FIT completion was not significantly different between participants in the large-declining and small-declining conditions (*p* = 0.069).

## Discussion

The results of our field experiment demonstrate that specifying a deadline in FIT invitations increases completion. Specifically, we found CRC screening was significantly higher when FIT invitations included *any* deadline—short, long, and both with and without a declining financial incentive tied to it—relative to a standard invitation. Moreover, as anticipated, we found that shorter deadlines more effectively increased FIT completion than longer deadlines—particular in the short-term. Most notably, our data suggest imposing a short deadline was as effective at encouraging FIT completion as a deadline coupled with a declining financial incentive.

Past research suggests that procrastination is particularly prevalent for aversive tasks, such as unpleasant medical procedures^15^. Given the aversive nature of FITs^11^, the finding that people delay completion should thus come as little surprise^11,12^. Procrastination begets procrastination, often leading to failure to complete a task altogether^14,20,24^. In the context of FITs, putting off the test until a future day could result in failure to complete screening entirely. Notably, we designed our interventions based on previous literature implicating procrastination as a key barrier to FIT completion^11–13^ and highlighting deadlines’ potential to curb procrastination. At the same time, we do not directly test procrastination in our data. Thus, any proposition we make in this article concerning the role of procrastination in FIT non-completion—and that of deadlines in attenuating procrastination—remains speculative. However, given the importance of screening in cancer detection and prevention^5^, and patients’ awareness of this importance as a result of previous knowledge, communication with their doctors, or reading our invitation, failing to complete screening acts against their interests. For these reasons, we postulate that it is reasonable to regard participants’ choice to delay FIT completion as procrastination even without direct documentation of intention.

Although suppositional, our findings offer two observations in support of our proposition that our interventions increased FIT completion by reducing procrastination. First, consistent with past research showing that imposing deadlines attenuates procrastination^18,29^, FIT completion in our experiment was significantly higher when invitations included a deadline, relative to when there was no deadline. Second, we found that adding a declining incentive to a deadline did not significantly increase completion beyond a short deadline with no financial incentive. This finding suggests that, at least in our study’s settings, targeting individuals’ procrastination tendencies by imposing a deadline could be as effective as adding extrinsic incentives^46^. Of note, because our incentive treatments both involved declining-incentive paradigms, we cannot speak to the effectiveness of a short deadline relative to a deadline with a fixed incentive. However, based on previous findings that declining incentives can be more motivating than fixed incentives^45^, we predict that adding a fixed incentive to a deadline would have a smaller effect than a declining incentive. In other words, comparing a non-incentivized deadline with a declining-incentive deadline was a more conservative test of our hypothesis.

We predicted participants in both declining-incentive conditions would be motivated by a desire to not miss out on the earlier, larger reward—as opposed to responding to the value of the incentive itself. Consequently, we anticipated completion rates would not differ across these conditions. However, our data show participants were more likely to complete the FIT in response to the larger-declining incentive than the smaller-declining incentive. Although this result could suggest larger incentives may be more effective at increasing FIT completion than smaller incentives, further examination would be necessary to determine the cost effectiveness of such an approach—especially given that we found a short deadline was as effective as a deadline coupled with declining incentives.

The present research provides the first empirical investigation, to our knowledge, comparing the effectiveness of non-incentivized deadlines versus incentivized deadlines on encouraging a targeted behavior. Our findings offer several important theoretical contributions. First, we contribute to the health, psychology, and economics literature by demonstrating that a short deadline can be as effective at encouraging certain behaviors (here, mailed FIT completion) as a deadline coupled with a declining financial incentive. By extension, we believe it is reasonable to expect that, in certain contexts, deadlines can be as effective as “regular” (i.e., non-declining) financial incentives. Second, we contribute to the cancer-screening literature, as this is the first experimental study to directly test whether deadlines increase FIT completion. We find that introducing deadlines, particularly short ones, increases screening completion—with or without a financial incentive tied to it. This finding is particularly intriguing given our study population—low-income individuals for whom $20, $10, or even $5 could potentially be impactful. Of note, although our study focused on FIT screening, it is reasonable to expect that the results of our field experiment would apply to other medical procedures in general, and other forms of cancer screenings in particular, that share key properties with FIT from patients' standpoint (e.g., being aversive in nature and/or being perceived as involving greater costs than benefits). Future research is necessary to test this assertion. Finally, we contribute to the literature on incentives by investigating a novel incentive structure that has the potential to increase the perceived value of an incentive by (1) specifying an expiration date (short, in particular), and (2) including a smaller incentive for reference comparison.

These findings also have important implications for clinical practice and public health interventions focused on promoting cancer screening, by providing insights about how to design effective interventions. Our results suggest that creating a limited time window is as effective at increasing CRC screening as other, costlier interventions, such as offering financial incentives. From a practical standpoint, this result is particularly important in light of the overwhelming increase in programs offering financial incentives in an effort to improve healthcare outcomes^47–49^. As an example, among large employers nationally, the per-employee wellness program expense increased from $260 in 2009 to $762 in 2019^50^. Recently, incentives have become center stage as federal and state governments, as well as healthcare providers and organizations, have been frantically trying to increase COVID-19 vaccine uptake^51,52^. While the effectiveness of these particular incentive campaigns is pending (most are still ongoing), it is clear they are financially costly. Further, research suggests that offering a large incentive, such as Governor Newsom’s plan to award ten California residents who get vaccinated $1.5 M each, could lead to unintended consequences such as increased skepticism and distrust^53^. Our findings suggest that imposing deadlines on vaccines, thus creating a sense of urgency and/or scarcity, may be a worthwhile intervention to consider. In certain contexts, providing financial incentives to encourage a behavior can be effective, such as when motivation is lacking^46^. Notably, however, the results of our field experiment—that short non-incentivized deadlines are as effective as deadlines coupled with financial incentives—highlight the value of first identifying the barriers associated with the focal task completion, as well as their relative importance, prior to launching an intervention. These insights should then serve as the starting point in any program design.

Finally, building on previous research on procrastination^16–19,24,29^, we cautiously speculate that our findings suggest that targeting procrastination tendencies may prove effective in promoting behaviors across a wide range of health-related (and other) tasks, especially when the costs associated with them are more salient than the benefits. From a health-outcomes perspective, our research offers a straightforward intervention to increase response and encourage quicker completion of CRC screening, at a lower cost, with the potential to improve individual and population-level health and well-being.

This study has several limitations of note. First, while FIT addresses some of the reasons people fail to complete CRC screening (e.g., difficulty attending an in-person procedure or fear of colonoscopy), failure to complete FIT is still multiply determined. In addition to task aversiveness and procrastination tendencies, researchers have proposed a host of other reasons for non-completion, including but not limited to, avoidance due to inattention, discussions (or lack thereof) with one’s doctors, and the belief screening is unnecessary^11,13^. Given the high proportion of patients who failed to complete FIT in our study, we suspect that even if procrastination impedes completion, it is likely just one of many reasons. In this research, we based our intervention design on previous research suggesting procrastination contributes to low FIT completion. However, as mentioned, we do not directly test procrastination in this study, and it is possible deadlines increased completion for other reasons. In addition, while deadlines increased completion, there are still many other barriers to completion that deadlines cannot address. For this reason, multipronged approaches are likely necessary to further encourage participation in such programs. Relatedly, behavioral barriers to FIT screening (such as procrastination) have not been well studied among this population. As such, more work is needed to understand whether procrastination is an important challenge to screening completion, and whether interventions focused on addressing procrastination (as opposed to focusing on other barriers, such as health insurance costs) are necessary. We acknowledge an additional limitation of this work regarding informed choice. Specifically, we did not collect data on whether the intervention material led to well informed choices to participate or not participate, and whether the financial incentives, though quite modest, could have been coercive such that some patients made an uninformed choice to participate in cancer screening. Future work could investigate this potential consequence.

In sum, the results of our field experiment show that imposing a deadline for CRC screening significantly increases completion relative to standard outreach, and that shorter deadlines are more effective than longer deadlines. Importantly, our data suggest increasing the appeal of the invitation by adding a declining financial incentive does not improve its effectiveness above and beyond a short deadline. These findings contribute to our understanding of the barriers to CRC screening, and potentially to other aversive health behaviors, and offer a low-cost intervention to increase response and encourage more timely completion.

## Materials and methods

Our field experiment evaluated the effectiveness of different mailed invitations to complete FITs among a low-income population not up to date with CRC screening. Enrollment began in September 2017; data collection ended in August 2018. The study’s protocol was approved by the UT Southwestern Medical Center (STU 012016-034) and John Peter Smith (JPS; 060616.003f.) Institutional Review Boards. Study approval included the waiver of informed consent and all methods were performed in accordance with relevant guidelines and regulations. The study was prospectively registered with ClinicalTrials.gov (NCT03181334).

### Participants and procedures

Individuals were eligible to participate if they were between the ages of 50 and 74, were part of the JPS Health Network, were uninsured or underinsured, spoke English or Spanish, had no history of colon cancer, colon resection, or inflammatory bowel disease, had not completed a colonoscopy in the past 10 years, a sigmoidoscopy in the last 5 years, or fecal occult blood test (FOBT) or fecal immunochemical test (FIT) in the last year, their complete contact information was on file, and they were not incarcerated or homeless.

Based on prior studies with this population, we assumed a return rate of 36.5% in the control group. We estimated needing 1026 participants per condition to detect at least an 8% absolute difference in the FIT completion rate between the five intervention groups. Patients were identified as being eligible for screening participation based on pre-established inclusion/exclusion criteria, using data extracted from the health system’s electronic medical record system. Accordingly, 7760 patients identified as eligible were randomized to one of five conditions within SQL to achieve 80% power to detect at least an 8% difference between groups when using a chi-square test with a two-sided Bonferroni corrected significance level of 0.005 (= 0.05/10). After randomization, but prior to analysis, 49 patients who were enrolled in a separate FIT outreach initiative—and were thus ineligible to participate in the trial and had been incorrectly randomized—were excluded. The 7711 eligible participants were randomly assigned to: (1) control (standard outreach; *n* = 1544); (2) short-deadline (1 week;* n* = 1542); (3) long-deadline (3 weeks; *n* = 1544); (4) small-declining ($10 for completing FIT within 1 week or $5 for completing it within 3 weeks; *n* = 1534); or (5) large-declining ($20 for completion within 1 week or $10 for completion within 3 weeks; *n* = 1547). The study was granted a waiver of informed consent through the IRB, as such blinding at the patient level was not performed (patients were unaware they were participating in a field experiment with different conditions). The program manager and navigation team were not blinded to assignment as they did not engage with study participants within the intervention period. The investigators and data analysts, however, were blinded throughout the randomization, data collection, and analysis process.

It is worth noting that the mailed FIT response rate in all study groups was lower than has been previously reported in other studies. For example, whereas mailed FIT response was 9.1% in the control condition in this study, it was previously reported to be 36.2% in a similar mailed FIT intervention lead by our group. We postulate several factors that might account for these lower observed response rates. In the prior work, branding of mailed FIT materials was based on the John Peter Smith Health System, whereas in the current experiment it was by Moncrief Cancer Institute (MCI). JPS was the medical home for our outreach participants and is well known throughout the community as it has been a fixture health care provider since 1906, and the only public hospital system in Tarrant county and Fort Worth, Texas. Moncrief Cancer Institute was established in 2006. It was previously named the Moncrief Radiation Center, before it took on an expanded role in care across the cancer care continuum, including for cancer prevention services. As such, it is possible that MCI, including its mission of cancer prevention, was less well known in the community and that this may have led to more discarded invitations and lower mailed FIT return. Additionally, the group not up to date with screening (and thus invited to be screened) might have been somewhat more refractory than prior studies, given time trends in CRC screening awareness, and also, prior implementation of interventions to promote CRC screening uptake in this population. Taken together—though random group assignment reduces the chances of unequal distribution of measurable and unmeasurable confounders between groups, and provides reassurance that the differences in response between groups are due to group-based differences in intervention features—the lower overall response rates observed across all intervention groups compared to previous studies of mailed outreach underscores a need for further research to confirm the potential utility of deadlines for improving completion of mailed FIT and other healthy behaviors.

We mailed all participants an invitation to complete the enclosed FIT and a 1-sample Polymedco OC Sensor FIT test. In addition, participants received two automated telephone reminders in English and Spanish: one at the time invitations were mailed and another a week later. Instructions and phone scripts are included in the Supplementary Materials. The letters addressed participants by their names and invited them to complete an at-home test to check for colon cancer. The letters also contained important facts about the test and informed participants that they were receiving the test at no cost to them. Participants wrote the date they collected their stool sample on the FIT vial prior to mailing. When the FIT was received, this date was entered into the database and used as the completion date in our analyses.

The treatment letters specified a date by which individuals were asked to return their kit. All dates included a three-day buffer to allow for mailing time. Thus, the short deadline (1 week) specified a date 10 days after the mail date; the long deadline (3 weeks) indicated a date 24 days after mailing. The text of the declining-incentive conditions informed participants they would receive a higher monetary reward if they returned their kit by a given date (10 days after mailing) or a smaller one if they returned it by a second, later date (24 days after mailing). All letters are presented in the Supplementary Materials. Among participants included in the primary analysis, 10 were mailed a second letter. Analyses were based on the initial mail date, and all 10 participants completed the FIT within 3 weeks of the initial invitation.

Participants who had an abnormal FIT result (≥ 100 ng hemoglobin/ml buffer and determined using the OC-Auto Micro 80 Analyzer) were contacted to schedule a follow-up diagnostic colonoscopy. For all participants, clinical services, including diagnostic colonoscopy where necessary, were provided at no cost. Participants with a normal FIT result, along with their primary care physician, were sent a result letter (participants eligible for a monetary reward also received a gift card with the appropriate amount). If any participants returned the FIT outside of the suggested time window, their test was still analyzed, and they were navigated to follow-up care, if necessary. Participants with a normal result were re-invited to participate in screening the subsequent year. The trial ended at 1 year, as was specified in the study protocol.

### Statistical analyses

Primary and secondary outcomes were evaluated using an intent-to-screen approach and included all participants. Analyses were conducted in R (version 4.0.2; R Foundation for Statistical Computing, Vienna, Austria, RRID: SCR_001905) and RStudio (version 1.3.1056; RStudio, Boston, MA, RRID: SCR_000432).

The preregistered primary outcome was the proportion of participants who completed the kit within 3 weeks of invitation. We chose the 3-week period for the primary analysis because it reflects the longest deadline given to participants. Further, 24 days after invitations were mailed, patients started receiving reminder calls on a rolling basis, which could have differentially influenced behavior. Thus, the 3-week window provides the cleanest test of our hypotheses. Within each condition, we calculated the proportion of returned kits using the number of participants who completed the FIT within 3 weeks as the numerator and all patients invited as the denominator. We used two-sided chi-square tests without Yates correction to compare the proportion of participants across conditions and significance was evaluated at the *p* = 0.005 level. For three participants, the completion date indicated on the returned sample was incorrect (the date recorded was prior to the invitation mail date). For these three observations (all received in under the 24-day period), we used the date the sample was received by the laboratory in lieu of the completion date written on the sample.

Although not preregistered, we also conducted secondary analyses to provide additional insights about the pattern of behaviors relevant to our primary outcome of interest (FIT completion within 3 weeks of invitation), and to assess whether our treatments affected behavior beyond the preregistered 3-week period. Secondary analyses examined: (1) completion likelihood within 1 week of invitation among those who completed the FIT within the 3-week window; (2) completion likelihood within 1 year of invitation (we opted for 1 year because eligible individuals should complete FITs annually); and, (3) average time to completion. To compare completion likelihood in the first week, we calculated the proportion of returned kits per condition using the number of participants who completed the FIT within 1 week as the numerator and those who completed it within 3 weeks as the denominator. To compare completion likelihood within 1 year, we calculated the proportion of returned kits per condition using the number of participants who completed the FIT prior to the data pull in September 2018 (1 year after invitation) as the numerator and all patients invited as the denominator. We used two-sided chi-square tests without Yates correction to compare the proportion of participants across conditions and significance was evaluated at the *p* = 0.005 level.

To calculate average completion time, we calculated the mean number of days between invitation and completion. We then used a Kruskal Wallis H test to assess whether the average number of days to completion differed across conditions. Finally, we used a post-hoc adjusted Wilcoxon rank sum test to conduct a pairwise comparison of the mean number of days between conditions.

## Data Availability

The data that support the findings of this study are available from UT Southwestern, but restrictions apply to the availability of these data, which were used under license for the current study, and so are not publicly available. Data are however available from the authors upon reasonable request and with permission of UT Southwestern.

## Code availability

The statistical code used is available from the corresponding author.
